# Isolation and Phylogenetic Analysis of *Parapoxvirus orf* From Outbreaks in Sheep in the Brazilian Midwest

**DOI:** 10.1111/zph.70081

**Published:** 2026-07-31

**Authors:** Phelipe Magalhães Duarte, Maria de Nazaré Santos Ferreira, Amanda Marques Cordeiro, Sérgio Alves do Nascimento, Rita de Cássia Carvalho Maia, José Wilton Pinheiro Junior

**Affiliations:** ^1^ Postgraduate Program in Animal Bioscience Federal Rural University of Pernambuco (UFRPE) Recife Pernambuco Brazil; ^2^ Instituto Aggeu Magalhães, FIOCRUZ Recife Pernambuco Brazil; ^3^ Animal Virology Laboratory, Department of Veterinary Medicine Federal Rural University of Pernambuco (UFRPE) Recife Pernambuco Brazil

**Keywords:** contagious ecthyma, epidemiology, one health, phylogenetics, viral

## Abstract

**Introduction:**

This study aimed to isolate *Parapoxvirus orf* and characterize its genetic diversity through phylogenetic analysis in small ruminants from the state of Mato Grosso, Brazil.

**Methods:**

A total of 103 scab samples were collected from sheep affected by nine contagious ecthyma outbreaks across three different mesoregions of Mato Grosso (Southeast, Northeast, and North) between 2023 and 2024. Viral DNA was amplified using conventional polymerase chain reaction (PCR) with primers PPP3 and PPP4 targeting the *B2L* gene to confirm the presence of *Parapoxvirus orf*. Following confirmation, 21 samples were selected for sequencing and phylogenetic analysis. Additionally, fifteen viable scab samples were selected for viral isolation using Madin–Darby bovine kidney cells cultured in RPMI medium in 24‐well plates at approximately 70% confluence 24 h after passage.

**Results:**

All scab samples (103/103) tested positive for *Parapoxvirus orf* by PCR. Phylogenetic analyses revealed genetic similarities between the obtained isolates and previously reported circulating isolates from Brazil, including a close genetic relationship with circulating isolates from Mato Grosso. Furthermore, a cytopathic effect was observed in 80% of the samples. PCR also detected *Parapoxvirus orf* in the culture supernatants from all isolation assays.

**Conclusion:**

This study confirms the circulation of *Parapoxvirus orf* in the state of Mato Grosso, Brazil. The genetic similarity of the identified isolates with those previously reported in Brazil and other countries highlights the need to strengthen biosecurity, prevention, and control policies for this zoonotic disease.

## Summary

- First large‐scale study of *Parapoxvirus orf* in sheep from the state of Mato Grosso, Brazil.
- Obtained isolates clustered closely with previously reported Mato Grasso isolates.
- Cytopathic effects occurred in 80% samples, including vacuole and syncytia formation.
- Findings support *Parapoxvirus orf* surveillance using the “One Health” approach.

## 1 Introduction

Contagious ecthyma is a zoonotic disease of occupational importance caused by *Parapoxvirus orf*, a DNA virus belonging to the family Poxviridae and genus *Parapoxvirus* (International Committee on Taxonomy of Viruses 2025; Kumar et al. 2014). *Parapoxvirus orf* is highly resistant in the environment and can remain viable for prolonged periods, especially under low‐temperature conditions (Duarte et al. 2026).

The *Parapoxvirus orf* genome consists of a linear double‐stranded DNA molecule approximately 138 kb in length with a high G+C content. It comprises a central conserved region flanked by two identical inverted terminal repeat sequences and harbours approximately 130–134 genes encoding a wide repertoire of viral proteins (Fleming et al. 2015).

Among these is the envelope protein gene *B2L*, which encodes the major viral envelope protein of approximately 42 kDa (Sullivan et al. 1994). *B2L* is widely used as a molecular marker for virus detection, molecular characterization, molecular epidemiology, and phylogenetic analyses (Abu Ghazaleh et al. 2023; Billinis et al. 2012; Milovanović et al. 2019; Oem et al. 2013).

The disease primarily affects sheep and goats, the natural hosts of *Parapoxvirus orf* (Fleming et al. 2015; Haig and Mercer 1998). However, rural workers, veterinarians, and other occupationally exposed individuals may become infected through direct contact with the infected animals, constituting an occupational risk group (Kumar et al. 2014; Veraldi et al. 2019).

In animals, contagious ecthyma typically affects the skin, lips, snout, nostrils, and oral mucosa, producing proliferative lesions with pustular and inflammatory characteristics (Haig and Mercer 1998). In humans, infection is generally self‐limiting and presents as nodular or papillomatous lesions, predominantly located on the hands and face (Hosamani et al. 2009).

Phylogenetic and molecular characterization studies based on sequence analysis have broadened our understanding of the genetic diversity and evolutionary dynamics of *Parapoxvirus orf*, thereby supporting the development of more effective diagnostic, prevention, and control strategies (Peralta et al. 2018). However, although contagious ecthyma has a worldwide distribution and outbreaks occur frequently, data on the genetic variability of *Parapoxvirus orf* remain scarce (Lojkic et al. 2010).

Therefore, this study aimed to isolate *Parapoxvirus orf* and investigate its phylogenetic relationships in small ruminants from the state of Mato Grosso, Brazil.

## 2 Materials and Methods

### 2.1 Sample Origin

A total of 103 samples were collected from nine outbreaks of contagious ecthyma in sheep. These outbreaks occurred in three mesoregions of the state of Mato Grosso, Brazil: Southeast (Rondonópolis and Campo Verde), Northeast (Barra do Garças), and North (Aripuanã, Guarantã do Norte, and Sinop), between 2023 and 2024 (Figure 1; Table 1).

**FIGURE 1:**
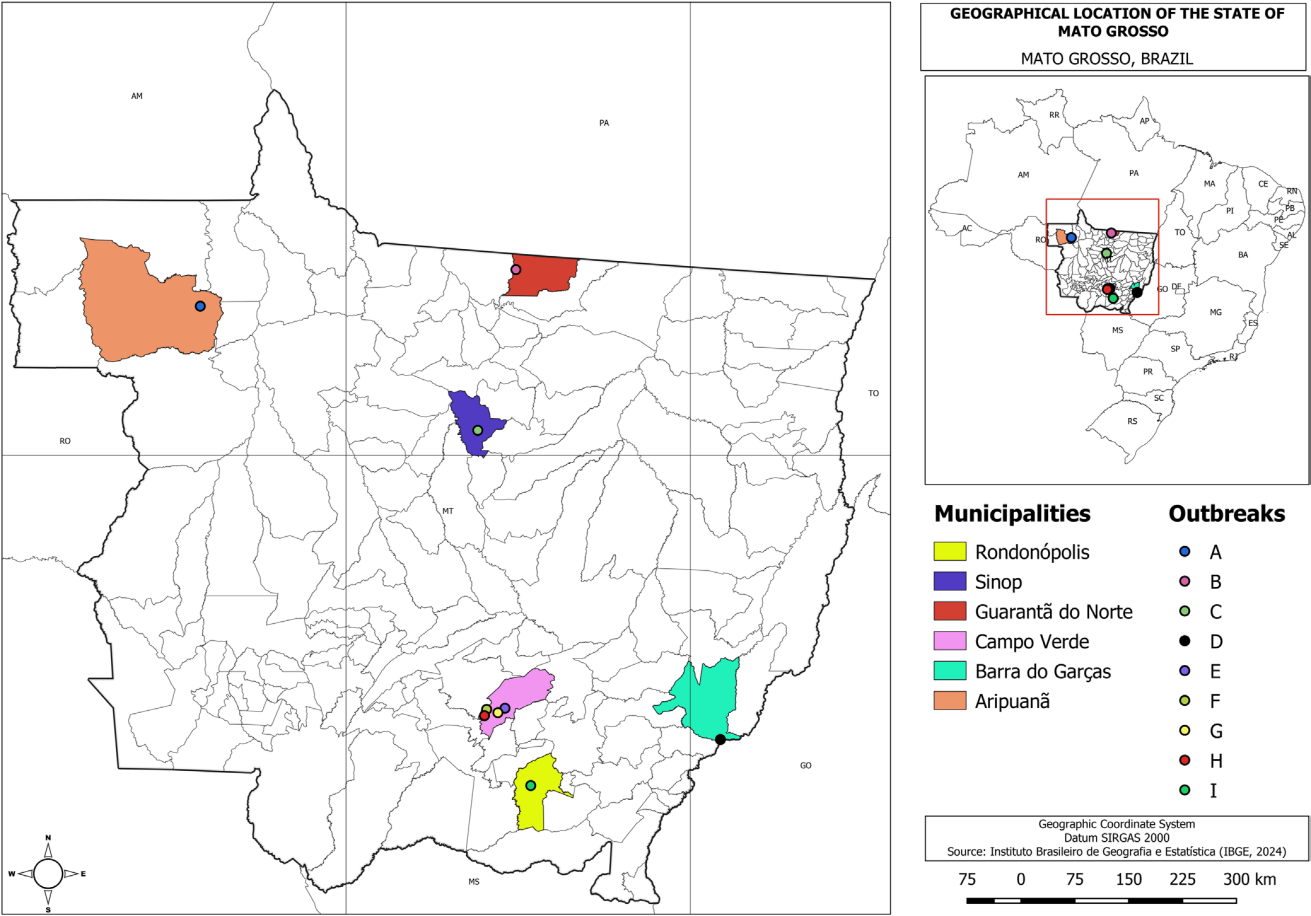
Geographic distribution of contagious ecthyma outbreaks in different municipalities of Mato Grosso, Brazil, during 2023–2024. Dots indicate the location and identification code of each outbreak.

**TABLE 1:** Data on the geographic location of outbreaks, number of outbreaks per municipality, outbreak classification, sample size, and identification of isolates obtained.

Mesoregion	Municipality	Outbreaks	Code	*n*	Isolated
North	Aripuanã	1	A	3	LAVIAN/MT/98—LAVIAN/MT/100
	Guarantã do Norte	1	B	49	LAVIAN/MT/49—LAVIAN/MT/97
	Sinop	1	C	1	LAVIAN/MT/101
Northeast	Barra do Garças	1	D	3	LAVIAN/MT/24—LAVIAN/MT/26
Southeast	Campo Verde	4	E	15	LAVIAN/MT/04—LAVIAN/MT/18
			F	5	LAVIAN/MT/19—LAVIAN/MT/21; LAVIAN/MT/102—LAVIAN/MT/103
			G	2	LAVIAN/MT/22—LAVIAN/MT/23
			H	22	LAVIAN/MT/27—LAVIAN/MT/48
	Rondonópolis	1	I	3	LAVIAN/MT/01—LAVIAN/MT/03

Approximately 5 g of scab material was collected from animals with clinical signs suggestive of contagious ecthyma using sterile scalpel blades and pre‐labelled 15‐mL Falcon tubes. Following sample collection, the lesions were treated with an iodine–glycerin solution or iodine solution. The samples were then refrigerated at 2°C–4°C during transport and stored in the laboratory at −20°C until processing.

### 2.2 Genomic DNA Extraction

To confirm the presence of *Parapoxvirus orf*, viral DNA was extracted from 25 mg of each scab sample using the Wizard SV Genomic DNA Purification System (Promega, United States of America [USA]), following the manufacturer's recommendations for tissue samples. The extracted DNA was subsequently quantified using the NanoDrop One^c^ (Thermo Fisher Scientific).

### 2.3 Polymerase Chain Reaction (PCR) and Sequencing

Viral genome was amplified by conventional PCR using a T100 Thermal Cycler (BIO‐RAD). Primers PPP3 and PPP4, which target an internal 235‐base pair (bp) fragment of the *B2L* gene, were used following the protocol established by Inoshima et al. (2000). The PCR protocol is summarized in Table 2.

**TABLE 2:** The PCR protocol adopted in this study for the identification of *Parapoxvirus orf*. The table shows the sequence of primers used to amplify the *B2L* gene.

Gene	Primer	Strand	Sequence	Size (pb)	Protocol	Reference
*B2L*	PP3	Forward	5′‐GCGAGTCCGAGAAGAATACG‐3′	235	Initial denaturation—3 min (94°C)	Inoshima et al. (2000)
	PP4	Reverse	5′‐TCAGTCGGAAGCGCCTCGCT‐3′		31 cycles: Denaturation—30s (94°C); Annealing—30s (60°C); Extension—1 min (72°C); Final Extension—1 min (72°C)	

The PCR products were analysed by electrophoresis on a 2% agarose gel (Sigma Aldrich) using 1000‐bp molecular size markers (Class Five, CV‐1003BP Ladder DNA). Positive and negative controls consisted of a previously sequenced *Parapoxvirus orf* isolate (LAVIAN/MT/01; GenBank accession number PX912061) and nuclease‐free water, respectively. The positive‐control sequence was as follows:

CGTGGCCGTGGGCGTGACGACGGCGCAGCAGAGCCGCGTGAACGGCACCTTCGGCTCCACGATCATGGAGTAAAAAGTGTTGTAGCGGTTCATGAGGTCCCAGGCCAGGTGCTTGTTGGTGGAGTAGAGCCC.

The amplified products were purified using the ReliaPrep DNA Clean‐Up and Concentration System (PROMEGA) according to the manufacturer's protocol. Subsequently, 21 PCR‐positive samples were selected for Sanger sequencing. At least one sample from each outbreak was included, with selection based on DNA quantity and viral genome composition with a predominance of conserved genes.

Sequencing was performed using the ABI BigDye Terminator Cycle Sequencing kit (Applied Biosystems), and the products were analysed using an ABI 3100 Genetic Analyser (Applied Biosystems).

### 2.4 Phylogenetic Analyses

Consensus sequences were generated using BioEdit (version 7.7.1), and for the construction of the database, 19 reference sequences were retrieved from the National Center for Biotechnology Information database (Table 3).

**TABLE 3:** Reference sequences obtained from the National Center for Biotechnology Information (NCBI) for database construction.

Identification	Host	Origin	Code	GenBank
Patos	Sheep	Brazil/PB	JX485993.1	*B2L*
São Martinho	Sheep	Brazil/RS	JX485980.1	*B2L*
Caprino_SJE	Goat	Brazil/PE	JX485992.1	*B2L*
Bahia	Goat	Brazil/BA	JX485987.1	*B2L*
Canguçú	Sheep	Brazil/RS	JX485989.1	*B2L*
MT05	Sheep	Brazil/MT	FJ665818.1	*B2L*
NE1	Sheep	Brazil/MT	FJ665819.1	*B2L*
SV547/11 Malhada	Dairy cows	Brazil/GO	JX268520.1	*B2L*
SV547‐11 Preta	Dairy cows	Brazil/GO	JX268519.1	*B2L*
LV 03/15	Sheep	Brazil/RS	MN524516.1	*B2L*
BR/UFBA/LabViro/Charles	Goat	Brazil/BA	KU310968.1	*B2L*
gLag14	Goat	Argentina	KY863425.1	*B2L*
UY09/79	Sheep	Uruguay	KP728922.1	*B2L*
gEma15	Goat	Argentina	KY863426.1	*B2L*
Malaysia/UPM‐2/14	Goat	Malaysia	KR024024.1	*B2L*
COVASPBN_GN_U_B2L_F_G11	Goat	India	PV116334.1	*B2L*
B032	Sheep	Germany	KF478797.1	*B2L*
CA47794b	Sheep	Italy	MW043025.1	*B2L*
Pseudocowpox virus strain PCPV776	Human	Austria	HM589036.1	*B2L*

Sequences from the 21 study samples were aligned using the Multiple Sequence Comparison by Log‐Expectation algorithm in the Molecular Evolutionary Genetics Analysis software version 12.0.

Evolutionary distances were calculated using the Maximum Likelihood method with 1000 bootstrap replicates in IQ‐TREE version 3.0.1 under the Jukes‐Cantor (JC) nucleotide substitution model, which was deemed the most appropriate given the low sequence divergence observed in the dataset. The resulting phylogenetic tree was visualized using the Interactive Tree of Life server (https://itol.embl.de/).

### 2.5 Viral Isolation in Cell Culture

All protocols and infrastructure complied with the biosafety requirements established by the Brazilian Ministry of Health for handling *Parapoxvirus orf* (Ministério da Saúde 2017).

A total of 15 samples were selected for viral isolation according to viable availability and the quantity required for each procedure.

For each sample, a 0.5 g tissue fragment was macerated and resuspended in 1–2 mL of RPMI medium supplemented with streptomycin (200 μg/mL) and penicillin G potassium (200 UI/mL) in a 15‐mL Falcon tube. The suspension was vortexed and centrifuged at 1680 × *g* for 10 min. The resulting supernatant (1 mL) was aspirated and transferred to a new sterile tube and centrifuged again at 4293 × *g* for 10 min. The final supernatant was used as the inoculum.

In this experiment, 24‐well plates containing Madin–Darby bovine kidney (MDBK) cells at approximately 70% confluence, 24 h after passage, were used for viral isolation.

Except for the negative controls, the culture medium from each well was aspirated and discarded. After discarding the medium, 400 μL of inoculum was added to each well and incubated for 1 h at 37°C and 5% CO_2_. The inoculum and the medium from the negative controls were then removed, and 2 mL of RPMI medium supplemented with 2% fetal bovine serum was added to each well. The plates were incubated at 37°C and examined for 7 days under an inverted microscope for cytopathic effects (CPE), which were graded as follows: + (≤ 25%); ++ (25%–50%); +++ (50%–75%); ++++ (75%–100%).

After 7 days, the plates underwent three freeze–thaw cycles to induce cell lysis and release a greater amount of viral particles into the supernatant for PCR analysis.

DNA was extracted from 150 μL of each culture supernatant using the Wizard Genomic DNA Purification System (Promega, USA), according to the manufacturer's protocol, and subsequently quantified. Viral DNA was then amplified by conventional PCR and the amplification products were analysed by agarose gel electrophoresis, following the same protocol described for the scab samples.

## 3 Results

### 3.1 PCR and Sequencing

All 103 sheep scab samples subjected to PCR tested positive for the *Parapoxvirus orf* (Figure 2).

**FIGURE 2:**
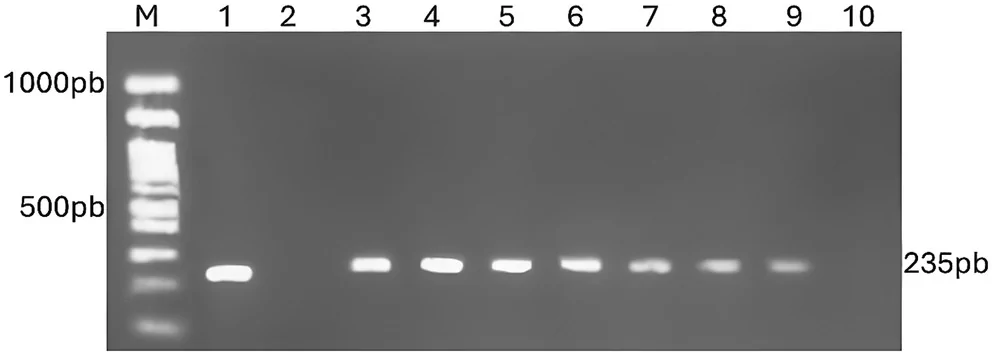
Agarose gel electrophoresis (2%) of polymerase chain reaction (PCR) products showing amplification of a 235‐bp fragment of the *Parapoxvirus orf B2L* gene from sheep scab samples. M, Molecular size marker (1000 bp); 1, positive control; 2, Negative control; 3–9, Positive samples; 10, Absent sample.

### 3.2 Phylogenetic Analyses

All sequenced samples were identified as *Parapoxvirus orf* belonging to the genus *Parapoxvirus* (Figure 3). The nucleotide sequences were deposited in GenBank under the accession numbers listed in Table 4.

**FIGURE 3:**
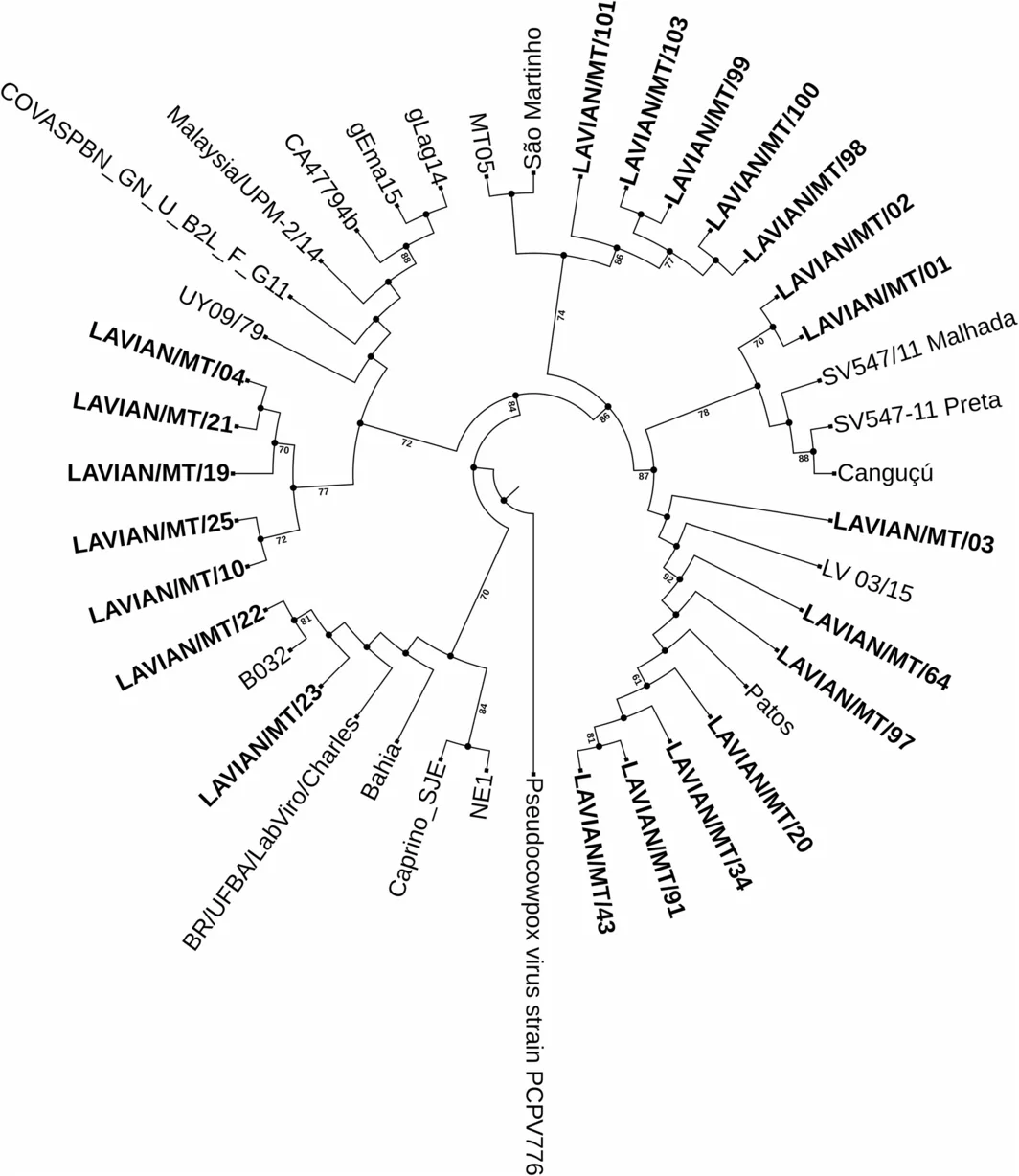
Maximum Likelihood phylogenetic tree based on partial *B2L* gene sequences of *Parapoxvirus orf* isolates obtained in this study and reference sequences retrieved from GenBank. Bootstrap values (1000 replicates) are shown at the branch nodes.

**TABLE 4:** List of sheep crust samples from Mato Grosso, Brazil, sequenced and deposited at the NCBI.

Identification	Host	Location	Code	GenkBank	Gene
LAVIAN/MT/01	Sheep	Rondonópolis/MT	I	PX912061	*B2L*
LAVIAN/MT/02	Sheep	Rondonópolis/MT	I	PX912062	*B2L*
LAVIAN/MT/03	Sheep	Rondonópolis/MT	I	PX912063	*B2L*
LAVIAN/MT/04	Sheep	Campo Verde/MT	E	PX912064	*B2L*
LAVIAN/MT/10	Sheep	Campo Verde/MT	E	PX912065	*B2L*
LAVIAN/MT/19	Sheep	Campo Verde/MT	F	PX912066	*B2L*
LAVIAN/MT/20	Sheep	Campo Verde/MT	F	PX912067	*B2L*
LAVIAN/MT/21	Sheep	Campo Verde/MT	F	PX912068	*B2L*
LAVIAN/MT/22	Sheep	Campo Verde/MT	G	PX912069	*B2L*
LAVIAN/MT/23	Sheep	Campo Verde/MT	G	PX912070	*B2L*
LAVIAN/MT/25	Sheep	Barra do Garças/MT	D	PX912071	*B2L*
LAVIAN/MT/34	Sheep	Campo Verde/MT	H	PX912072	*B2L*
LAVIAN/MT/43	Sheep	Campo Verde/MT	H	PX912073	*B2L*
LAVIAN/MT/64	Sheep	Guarantã do Norte/MT	B	PX912074	*B2L*
LAVIAN/MT/91	Sheep	Guarantã do Norte/MT	B	PX912075	*B2L*
LAVIAN/MT/97	Sheep	Guarantã do Norte/MT	B	PX912076	*B2L*
LAVIAN/MT/98	Sheep	Aripuanã/MT	A	PX912077	*B2L*
LAVIAN/MT/99	Sheep	Aripuanã/MT	A	PX912078	*B2L*
LAVIAN/MT/100	Sheep	Aripuanã/MT	A	PX912079	*B2L*
LAVIAN/MT/101	Sheep	Sinop/MT	C	PX912080	*B2L*
LAVIAN/MT/103	Sheep	Campo Verde/MT	F	PX912081	*B2L*

### 3.3 Viral Isolation in Cell Culture

Following viral isolation in cell culture, CPE were observed in 12/15 (80%) samples after 24 h of incubation at 37°C. Among these, 6/15 (40%) samples were graded as CPE +, 2/15 (13%) as CPE +++, and 4/15 (27%) as CPE ++++. Meanwhile, 3/15 (20%) samples showed absence of cytopathic effect (ACE).

The predominant CPE observed included vacuole formation, syncytia formation, cell degeneration, cell rounding, and cell death (Figure 4).

**FIGURE 4:**
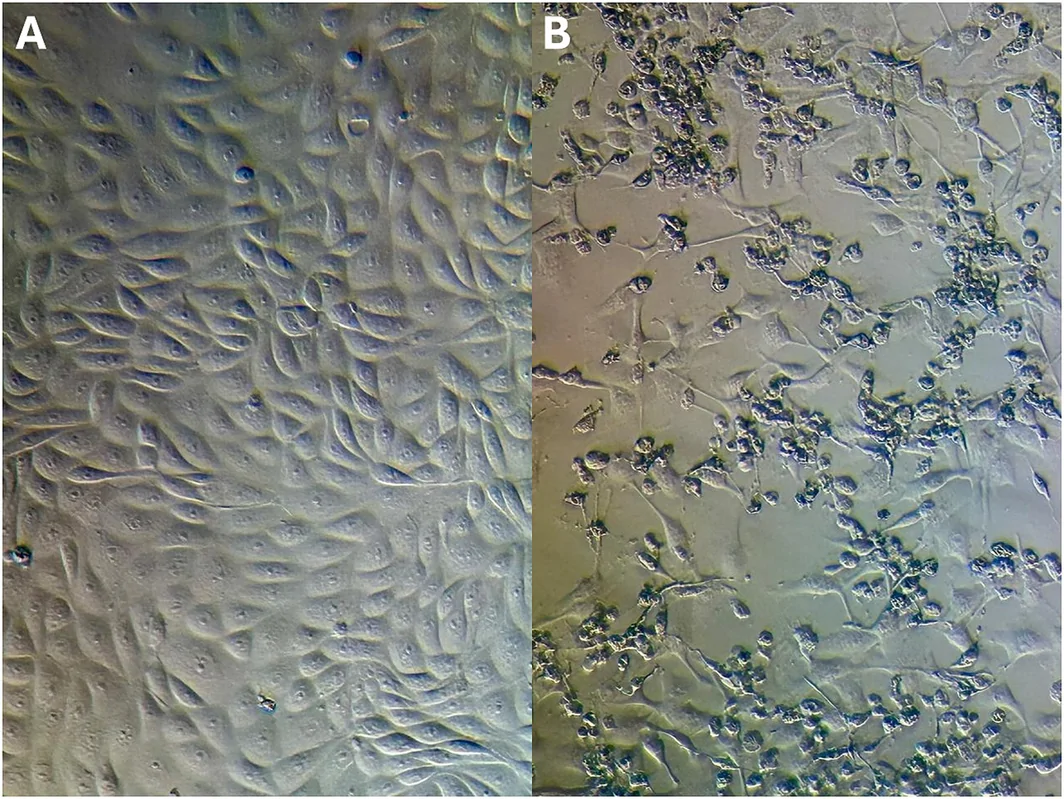
Cytopathic effects (CPE) induced by *Parapoxvirus orf* in Madin–Darby bovine kidney (MDBK) cells. (A) Uninfected MDBK cells (negative control); (B) infected MDBK cells showing CPE 24 h after infection (40× magnification).

All supernatant samples obtained following viral isolation tested positive for *Parapoxvirus orf* by PCR (15/15, 100%).

## 4 Discussion

In this study, all sequenced isolates were identified as *Parapoxvirus orf*. Detection of *Parapoxvirus orf* in all sheep scab samples, collected from outbreaks across multiple mesoregions, confirms the wide circulation of the virus in sheep in the state of Mato Grosso, Brazil, consistent with previous reports from the state (Abrahão et al. 2009).

The widespread detection of *Parapoxvirus orf* in Mato Grosso has several important implications. First, *Parapoxvirus orf* is a zoonotic pathogen that poses an occupational risk to individuals who have direct contact with infected animals, highlighting its relevance within the One Health framework.

Second, the continued circulation of the virus may threaten the expanding sheep and goat farming sector in the state, where herds are estimated at approximately 360,838 and 22,107 head, respectively (Instituto Brasileiro de Geografia e Estatística 2024a, 2024b), owing to the substantial economic losses resulting from contagious ecthyma (Moovendan et al. 2025).

Third, the wide geographic dispersion of the virus may indicate significant gaps in disease prevention and control programs, particularly inadequate prophylactic measures. These shortcomings may not only facilitate viral circulation but also reflect limited disease awareness among rural livestock producers and animal health professionals, thereby increasing the risk of zoonotic transmission.

Fourth, once introduced into the herd, the virus may become endemic (Smith and Sherman 1994) because of its prolonged environmental persistence (Duarte et al. 2026). Thus, the occurrence of outbreaks in multiple regions of Mato Grosso may indicate either endemic circulation or, in the case of primary outbreaks, ongoing establishment, which further reinforces the need for long‐term prevention and control strategies. However, epidemiological studies must be conducted to confirm the disease's endemicity.

Finally, no vaccine for contagious ecthyma is currently authorized for commercial use in Brazil, an aggravating factor that may contribute to continued viral circulation in domestic herds. Furthermore, autogenous vaccines remain underutilized in the region. Vaccination is an important disease‐control strategy for promoting health and productivity in small ruminants, while also reducing occupational exposure and the risk of infections in humans (Reichen et al. 2025).

Phylogenetic analyses revealed genetic similarities between the isolates obtained in this study and the *Parapoxvirus orf* isolates previously reported in Brazil between 2005 and 2015, including BR/UFBA/LabViro/Charles (Bahia), São Martinho (Rio Grande do Sul), SV547/11 Malhada (Goiás), SV547‐11 Preta (Goiás), Canguçú (Rio Grande do Sul), LV 03/15 (Rio Grande do Sul), Patos (Paraíba), Bahia (Bahia), and Caprino_SJE (Pernambuco). The isolates also showed high genetic similarity with circulating isolates previously reported in Mato Grosso (NE1 and MT05).

Furthermore, the isolates were genetically similar with *Parapoxvirus orf* isolates reported from Northeastern, Midwestern, and Southern Brazil (Abrahão et al. 2009; Schmidt et al. 2013). These findings reinforce the importance of strengthening epidemiological surveillance and biosecurity measures, particularly regarding the transit and free breeding of animals without appropriate inspection. Similarities with isolates from South America (Peralta et al. 2018), Europe (Coradduzza et al. 2021), and Asia (Abdullah et al. 2015) further demonstrate the broad geographic distribution of *Parapoxvirus orf* and its capacity for transboundary spread (Wang et al. 2019).

However, the isolates obtained in this study showed greater genetic proximity with each other than with geographically distant reference isolates. This pattern may also reflect the relatively conserved nature of the *Parapoxvirus orf* genome (Chen et al. 2017). Similar studies have been conducted in Italy (Coradduzza et al. 2021) and Brazil (Schmidt et al. 2013), both of which used partial sequencing of the *B2L* gene for the molecular characterization of *Parapoxvirus orf* obtained from small ruminants with clinical signs suggestive of contagious ecthyma. Nevertheless, because partial *B2L* sequencing provides limited phylogenetic resolution, further studies incorporating whole genome sequencing would improve evolutionary and phylogeographic analyses of *Parapoxvirus orf*.

Viral isolation remains the gold standard for diagnosing viral infections (Lemos et al. 2023). In the present study, CPE was observed in 12 of the 15 (80%) samples inoculated into MDBK cells. MDBK cells are widely used for isolating different viral pathogens, including *Parapoxvirus orf*, owing to their ease of maintenance and large‐scale cultivation (Inoshima et al. 1999). The CPE observed in the present study, such as cell rounding, degeneration, vacuole formation, and syncytium formation, was consistent with previous descriptions of *Parapoxvirus orf* infection in MDBK cells (Kruse and Weber 2001; Vikøren et al. 2008). The predominance of low CPE (+) with ACE (60% combined) in some cultures may reflect lower viral loads, thereby limiting the detection of CPE in the inoculated cells. Despite this, all culture supernatants tested positive for *Parapoxvirus orf* by PCR. By contrast, the occurrence of more pronounced CPE (+++ and ++++; 40% combined) may be associated with higher viral loads. In addition, the ovine species has high susceptibility to *Parapoxvirus orf* (Kroon et al. 2016).

Studies on the genetic diversity and infectivity of *Parapoxvirus orf* circulating in Brazilian sheep and goat populations remain lacking. Therefore, expanding molecular surveillance in different regions of Brazil may not only improve our knowledge of the epidemiology of contagious ecthyma, but also support the development of regionally appropriate vaccines and disease control strategies.

This study reports the first isolation of *Parapoxvirus orf* from sheep involved in nine contagious ecthyma outbreaks across northern, northeastern, and southeastern mesoregions of Mato Grosso, Brazil. The close genetic relationship between these isolates and previously reported Brazilian and international isolates highlights the importance of continued surveillance and strengthened biosecurity measures. Because contagious ecthyma is a zoonotic disease within the One Health framework, its prevention and control require a multidisciplinary and multisectoral approach. Future studies must adopt a comprehensive approach, based on broader research using complete genomes, to better elucidate the genetic variability among *Parapoxvirus orf* strains and to identify the key genes involved in viral immunomodulation.

## Funding

This work was supported by the Coordenação de Aperfeiçoamento de Pessoal de Nível Superior (CAPES), Brazil (Grant No 88887.854352/2023‐00).

## Ethics Statement

The study was approved by the Comissão de Ética no Uso de Animais of the Universidade Federal Rural de Pernambuco (CEUA/UFRPE) under approval number 8820160623 (ID 001491), in accordance with Resolution No. 436/2005. In addition, the study was duly registered with the Sistema Nacional de Gestão do Patrimônio Genético e do Conhecimento Tradicional Associado (SISGEN) under registration number A7E047D.

## Conflicts of Interest

The authors declare no conflicts of interest.

## Data Availability

The *Parapoxvirus orf* sequences obtained in this study were submitted to the National Center for Biotechnology Information (NCBI) and are publicly accessible under accession numbers PX912061 to PX912081.

## References

[zph70081-bib-0001] Abdullah, A. A. , M. F. Ismail , K. N. Balakrishnan , et al. 2015. “Isolation and Phylogenetic Analysis of Caprine Orf Virus in Malaysia.” Virusdisease 26: 255–259. 10.1007/s13337-015-0278-4. PMC4663714 26645035

[zph70081-bib-0002] Abrahão, J. S. , R. K. Campos , G. S. Trindade , et al. 2009. “Detection and Phylogenetic Analysis of Orf Virus From Sheep in Brazil: A Case Report.” Virology Journal 6: 47. 10.1186/1743-422X-6-47. PMC2685785 19413907

[zph70081-bib-0003] Abu Ghazaleh, R. , M. Al‐Sawalhe , I. Abu Odeh , J. El Ibrahim , B. Al‐Turman , and J. Makhamreh . 2023. “Host Range, Severity and Transboundary Transmission of Orf Virus (ORFV).” Infection, Genetics and Evolution 112: 105448. 10.1016/j.meegid.2023.105448. 37217030

[zph70081-bib-0004] Billinis, C. , V. S. Mavrogianni , V. Spyrou , and G. C. Fthenakis . 2012. “Phylogenetic Analysis of Strains of Orf Virus Isolated From Two Outbreaks of the Disease in Sheep in Greece.” Virology Journal 9: 24. 10.1186/1743-422X-9-24. PMC3275544 22260526

[zph70081-bib-0005] Chen, H. , W. Li , Z. Kuang , et al. 2017. “The Whole Genomic Analysis of Orf Virus Strain HN_3/12_ Isolated From Henan Province, Central China.” BMC Veterinary Research 13: 260. 10.1186/s12917-017-1178-1. PMC5562994 28821255

[zph70081-bib-0006] Coradduzza, E. , D. Sanna , A. M. Rocchigiani , et al. 2021. “Molecular Insights Into the Genetic Variability of ORF Virus in a Mediterranean Region (Sardinia, Italy).” Life (Basel, Switzerland) 11: 416. 10.3390/life11050416. PMC8147818 34064326

[zph70081-bib-0007] Duarte, P. M. , M. N. S. Ferreira , A. M. Cordeiro , et al. 2026. “Infectivity of Parapoxvirus Orf After 35 Years of Refrigeration.” Current Microbiology 83: 69. 10.1007/s00284-025-04659-3. 41369791

[zph70081-bib-0008] Fleming, S. B. , L. M. Wise , and A. A. Mercer . 2015. “Molecular Genetic Analysis of Orf Virus: A Poxvirus That Has Adapted to Skin.” Virus Genes 7: 1505–1539. 10.3390/v7031505. PMC4379583 25807056

[zph70081-bib-0009] Haig, D. M. , and A. A. Mercer . 1998. “Ovine Diseases. Orf.” Veterinary Research 29: 311–326. 9689744

[zph70081-bib-0010] Hosamani, M. , A. Scagliarini , V. Bhanuprakash , C. J. McInnes , and R. K. Singh . 2009. “Orf: An Update on Current Research and Future Perspectives.” Expert Review of Anti‐Infective Therapy 7: 879–893. 10.1586/eri.09.64. 19735227

[zph70081-bib-0011] Inoshima, Y. , A. Morooka , and H. Sentsui . 2000. “Detection and Diagnosis of Parapoxvirus by the Polymerase Chain Reaction.” Journal of Virological Methods 84: 201–208. 10.1016/S0166-0934(99)00144-5. 10680970

[zph70081-bib-0012] Inoshima, Y. , S. Shimizu , N. Minamoto , K. Hirai , and H. Sentsui . 1999. “Use of Protein AG in an Enzyme‐Linked Immunosorbent Assay for Screening for Antibodies Against Parapoxvirus in Wild Animals in Japan.” Clinical and Diagnostic Laboratory Immunology 6: 388–391. 10.1128/CDLI.6.3.388-391.1999. PMC103728 10225841

[zph70081-bib-0013] Instituto Brasileiro de Geografia e Estatística . 2024a. “Rebanho de ovinos (ovelhas e carneiros).” IBGE. https://www.ibge.gov.br/explica/producao‐agropecuaria/ovino/mt.

[zph70081-bib-0014] Instituto Brasileiro de Geografia e Estatística . 2024b. “Rebanho de caprinos.” IBGE. https://www.ibge.gov.br/explica/producao‐agropecuaria/caprinos/mt.

[zph70081-bib-0015] International Committee on Taxonomy of Viruses . 2025. “Current ICTV Taxonomy Release – Taxonomy Browser.” https://ictv.global/taxonomy.

[zph70081-bib-0016] Kroon, E. G. , Z. I. P. Lobato , and M. I. M. C. Guedes . 2016. “Enfermidades causadas por Orthopvirus e Parapoxvirus. Ectima contagioso.” In Doenças infecciosas em animais de produção e de companhia, edited by J. Megid , M. G. Ribeiro , and A. C. Paes , 642–650. Roca.

[zph70081-bib-0017] Kruse, N. , and O. Weber . 2001. “Selective Induction of Apoptosis in Antigen‐Presenting Cells in Mice by Parapoxvirus Ovis.” Journal of Virology 75: 4699–4704. 10.1128/JVI.75.10.4699-4704.2001. PMC114224 11312341

[zph70081-bib-0018] Kumar, N. , A. Wadhwa , K. K. Chaubey , et al. 2014. “Isolation and Phylogenetic Analysis of an Orf Virus From Sheep in Makhdoom, India.” Virus Genes 48: 312–319. 10.1007/s11262-013-1025-9. 24347045

[zph70081-bib-0019] Lemos, E. R. S. , L. M. Villar , L. A. A. Leon , M. L. Guimarães , and S. L. M. Teixeira , eds. 2023. Tópicos em virologia. Editora Fiocruz.

[zph70081-bib-0020] Lojkic, I. , Z. Cac , A. Beck , T. Bedekovic , Z. Cvetnic , and B. Sostaric . 2010. “Phylogenetic Analysis of Croatian Orf Viruses Isolated From Sheep and Goats.” Virology Journal 7: 314. 10.1186/1743-422X-7-314. PMC2989325 21073725

[zph70081-bib-0021] Milovanović, M. , V. Milićević , M. Valčić , N. Stević , J. Nišavić , and S. Radojičić . 2019. “Detection and Phylogenetic Analysis of B2L Gene of ORF Virus From Clinical Cases of Sheep in Serbia.” Pakistan Veterinary Journal 39: 433–437. 10.29261/pakvetj/2019.034.

[zph70081-bib-0022] Ministério da Saúde . 2017. “Classificação de risco dos agentes biológicos.” https://pncq.org.br/uploads/classificacao_risco_agentes_biologicos_3ed.pdf.

[zph70081-bib-0023] Moovendan, M. , S. Vivek , K. Rajkumar , et al. 2025. “Economic Impact of an ORF Outbreak in Puducherry Region.” International Journal of Veterinary Sciences and Animal Husbandry 10: 10–15. 10.22271/veterinary.2025.v10.i5Sa.2282.

[zph70081-bib-0024] Oem, J. K. , J. Y. Chung , Y. J. Kim , et al. 2013. “Isolation and Characterization of Orf Viruses From Korean Black Goats.” Journal of Veterinary Science 14: 227–230. 10.4142/jvs.2013.14.2.227. PMC3694196 23814477

[zph70081-bib-0025] Peralta, A. , C. A. Robles , J. F. Micheluod , et al. 2018. “Phylogenetic Analysis of ORF Viruses From Five Contagious Ecthyma Outbreaks in Argentinian Goats.” Frontiers in Veterinary Science 5: 134. 10.3389/fvets.2018.00134. PMC6018470 29971243

[zph70081-bib-0026] Reichen, C. , B. C. B. Beirão , and A. L. G. Monteiro . 2025. “Contagious Ecthyma in Small Ruminants: From Etiology to Vaccine Challenges ‐ A Review.” Veterinary Research Communications 49: 115. 10.1007/s11259-025-10677-0. 39992468

[zph70081-bib-0027] Schmidt, C. , J. F. Cargnelutti , M. C. Brum , C. K. Traesel , R. Weiblen , and E. F. Flores . 2013. “Partial Sequence Analysis of B2L Gene of Brazilian Orf Viruses From Sheep and Goats.” Veterinary Microbiology 162: 245–253. 10.1016/j.vetmic.2012.10.031. 23200438

[zph70081-bib-0028] Smith, M. C. , and D. M. Sherman , eds. 1994. Goat Medicine, 535–540. Lea and Febiger.

[zph70081-bib-0029] Sullivan, J. T. , A. A. Mercer , S. B. Fleming , and A. J. Robinson . 1994. “Identification and Characterization of an Orf Virus Homologue of the Vaccinia Virus Gene Encoding the Major Envelope Antigen p37K.” Virology 202: 968–973. 10.1006/viro.1994.1420. 8030257

[zph70081-bib-0030] Veraldi, S. , L. Esposito , P. Pontini , F. Vaira , and G. Nazzaro . 2019. “Feast of Sacrifice and Orf, Milan, Italy, 2015–2018.” Emerging Infectious Diseases 25: 1585–1586. 10.3201/eid2508.181063. PMC6649321 31310206

[zph70081-bib-0031] Vikøren, T. , A. Lillehaug , J. Akerstedt , T. Bretten , M. Haugum , and M. Tryland . 2008. “A Severe Outbreak of Contagious Ecthyma (Orf) in a Free‐Ranging Musk Ox ( *Ovibos moschatus* ) Population in Norway.” Veterinary Microbiology 127: 10–20. 10.1016/j.vetmic.2007.07.029. 17768017

[zph70081-bib-0032] Wang, Y. , K. Yang , Y. A. Wang , et al. 2019. “Identification and Phylogenetic Analysis of an Orf Virus Strain Isolated in Anhui Province, East‐Central China, in 2018.” Acta Virologica 63: 270–277. 10.4149/av_2019_304. 31507192

